# Hospital Plans and Their Selection

**Published:** 1914-06-27

**Authors:** 


### HOSPITAL PLANS AND THEIR SELECTION.

### Right and Wrong Methods: A Case in Point.

For twenty years we have continuously urged the importance of associating a practical hospital administrator of proved knowledge with each architect who may be charged with the duty of preparing plans for a new hospital, or with some material reconstruction scheme or additions to existing buildings. Unfortunately, in this country there is at present no adequate system to secure the selection by competition of the best plan submitted, much less to provide hospital committees with a guarantee that the new hospital, or the new department of a hospital, when erected will be thoroughly up-to-date, modern, and complete in every department. One frequent cause of the failures of which we have had several examples in recent years is due to the fact that the power to decide is left to one assessor. This assessor, whoever he may be, must often fail for the reason that the modern hospital is many-sided, and that few or no architects by themselves have the technical knowledge to enable them to judge which out of many plans, so far as the administrative and other requirements of a modern hospital are concerned, is, in fact, the best. Another cause of failure is that committees have not yet realised the importance of associating with any architect assessor a competent hospital expert who is admittedly an authority on the many technical and administrative matters which must materially govern a just and satisfactory decision as to the respective merits of a number of plans for a new hospital or new buildings submitted in competition. Some voluntary hospitals have a competent expert on their staff, whom they should encourage and trust.

A further step against reform is now being attempted by some architect assessors, we understand, who make it a condition that any decision they may arrive at in regard to sets of plans sent in in competition shall be final, and that any set they may select shall be adopted and carried out by the hospital authorities. We regard this claim as preposterous and dangerous, because the perfect architect assessor has never made himself manifest in this country, at any rate, and no committee, holding its responsibilities, as all do, in trust for the subscribers and for the people of a given community, can properly, or even wisely, accept an assessor who insists upon being first placed in the position of an autocrat, and then proceeding to judge (?) the plans of other architects submitted in competition.

We hear that the Royal Infirmary, Liverpool, has before it the duty of making plans and securing the speedy erection of a perfect, up-to-date home for its nurses. In our report on this infirmary* we declared with justice that, by his thoroughness, the late Mr. Alfred Waterhouse, R.A., the architect to this hospital, though the buildings are upwards of twenty years old, has placed his creation "deservedly first amongst the voluntary hospital buildings in this part of the country." This fact makes the procedure to be adopted by the committee of the Liverpool Royal Infirmary in the preparation and settlement of the plans for its new nurses' home a matter of deep interest to hospital managers throughout the country. The eye of the hospital world is concentrated on the managers, and their decision is awaited with the keenest interest, because they have an unique opportunity to exhibit courage and do the right thing in the right way.

What the hospital world is waiting to see is whether the committee are aware of the technical force which their team of officer experts represents, and whether they will, in a relatively simple question like that of a nurses' home, instruct their officers first to prepare for their consideration pencil drawings and a practical knowledgeable scheme.

The infirmary is blessed with an influential, competent, and sound man of business in its present chairman, Mr. J. Wade Deacon, whom we congratulate upon his appointment as chairman of the British Hospitals Association. He is a man of sober judgment, and he must be quite aware that the procedure to be followed, and the actual planning of the nurses' home, together with the method of securing a good one, materially influence the ease or difficulty he will experience in raising the necessary £30,000 to defray the cost of its erection.

Looking to the proud position occupied by the Royal Infirmary in regard to buildings, at the proved competence and knowledge of the two officials most interested in the efficiency of the plan and arrangements for the new nurses' home —*i.e.* the general superintendent and the matron—and strengthened by the splendid results exhibited by the new out-patient department—the plans for which were evolved by following the practical methods we are here discussing—there can be no doubt that the wisest course would be to adopt the business method of entrusting these two officials, with the chairman, in conjunction with a competent architect like Mr. Paul Waterhouse, who has worthily displayed much of his father's ability in hospital construction, with the preparation of plans for the nurses' home. This small group of experts would prepare and submit a scheme with full details for the consideration of the committee in the first instance. Here we may say that in a great institution like this the expenditure of £30,000 upon buildings is a relatively small matter which does not justify an elaborate and costly scheme of competitive plans by a number of architects, because the cost would be out of all proportion to any good results which could possibly flow from such a course. It is not likely that a limited competition for a nurses' home, of which very few members of the architectural profession

* The Hospital, January 8, 1910, pp. 425-427.

have any experience, would produce a satisfactory plan. In such circumstances to appoint an architect assessor, and to invite a number of architects, who could not possess adequate knowledge of the requirements of the infirmary work, staff, and circumstances, would be to court disappointment and, in view of past competitions, almost certain failure too. On the other hand, the alternative and relatively costless plan which we have suggested guarantees that the new nurses' home so created, based as it would be upon practical knowledge and a full apprehension of all that is required at Liverpool in regard to the new home, should at least prove worthy of the excellent units and buildings already possessed by the managers of the Royal Infirmary, Liverpool.

We have every confidence that the Chairman and committee of the Liverpool Royal Infirmary will rise above all local prejudices and unite as men of business to follow the simple, practical course which they are fortunate enough to be in a position to adopt, and which is demonstrably the best.

